# ﻿Three new species of the planthopper genus *Oecleopsis* Emeljanov, 1971 from China (Hemiptera, Fulgoromorpha, Cixiidae)

**DOI:** 10.3897/zookeys.1188.114008

**Published:** 2024-01-10

**Authors:** Sha-Sha Lv, Lin Yang, Yu-Bo Zhang, Yan Zhi, Pei Zhang, Xiang-Sheng Chen

**Affiliations:** 1 Institute of Entomology, Guizhou University, Guiyang, Guizhou, 550025, China; 2 The Provincial Special Key Laboratory for Development and Utilization of Insect Resources of Guizhou, Guizhou University, Guiyang, Guizhou, 550025, China; 3 Anshun University, College Agriculture, Anshun, Guizhou, 561000, China; 4 Laboratory Animal Center, Guizhou Medical University, Guiyang, Guizhou, 550025, China; 5 Xingyi Normal University for Nationalities, Xingyi, Guizhou, 562400, China

**Keywords:** Distribution, Fulgoroidea, morphology, Pentastirini, taxonomy

## Abstract

Three new species of the genus *Oecleopsis* Emeljanov, 1971 from China, *O.acerbus* Lv & Chen, **sp. nov.** and *O.panxianensis* Lv & Chen, **sp. nov.** from Guizhou Province, and *O.digitatus* Lv & Chen, **sp. nov.** from Sichuan Province, are described and illustrated. With these additions, the number of species in the genus is increased to 18. An updated identification key and checklist of all known species of *Oecleopsis* are provided as well as a map of their geographic distributions.

## ﻿Introduction

Emeljanov (1971) established the planthopper genus *Oecleopsis* with the type species *Oecleopsisartemisiae* (Matsumura, 1914) and transferred *O.cucullatus* (Noualhier, 1896) from the genus *Oliarus* Stål, 1862 into the genus *Oecleopsis*. This genus belongs to the tribe Pentastirini of the subfamily Cixiinae (Hemiptera, Fulgoromorpha, Cixiidae) (Emeljanov 1971; Bourgoin 2023). Van Stalle (1991) described *O.articara* Van Stalle, 1991, and transferred the following seven species from *Oliarus* Stål, 1862 to *Oecleopsis*: *O.petasatus* (Noualhier, 1896), *O.mori* (Matsumura, 1914), *O.sinicus* (Jacobi, 1944), *O.yoshikawai* (Ishihara, 1961), *O.bifidus* (Tsaur, Hsu & Van Stalle, 1988), *O.chiangi* (Tsaur, Hsu & Van Stalle, 1988), and *O.elevatus* (Tsaur, Hsu & Van Stalle, 1988). Guo et al. (2009) reviewed the genus and described three new species from China, *O.spinosus* Guo, Wang & Feng, 2009, *O.tiantaiensis* Guo, Wang & Feng, 2009, and *O.wuyiensis* Guo, Wang & Feng, 2009. Zhi et al. (2018) described two new species from China, *O.laminatus* Zhi & Chen, 2018 and *O.productus* Zhi & Chen, 2018. Until now, 15 species have been recorded in the genus, which are widely distributed in the Palaearctic (China, Japan, Russia, and Korea) and Oriental (China, Cambodia, Indonesia, Malaysia, and Thailand) regions (Holt et al. 2013; Bourgoin 2023).

Herein, three new species from China, *O.acerbus* Lv & Chen sp. nov., *O.panxianensis* Lv & Chen, sp. nov. and *O.digitatus* Lv & Chen, sp. nov., are described and illustrated. Hence, the species number of *Oecleopsis* is raised to 18. All *Oecleopsis* species are recorded from China (Guo et al. 2009; Zhi et al. 2018; Luo et al. 2022; Bourgoin 2023).

## ﻿Material and methods

The external morphology terminologies are as follows: male genitalia follows Bourgoin (1987), female genitalia follows Bourgoin (1993), and wing venation follows Bourgoin et al. (2015). Body measurements are from apex of vertex to tip of forewing; vertex length was measured the median length of vertex (from apical transverse carina to tip of basal emargination). All measurements are in millimeters (mm). External morphology and drawings were done under the Leica MZ 12.5 stereomicroscope. Photographs were taken with NIKON SMZ 25 and VHX-1000E digital camera. Illustrations were scanned with CanoScan LiDE 200 and imported into Adobe Photoshop 7.0 for labeling and plate composition. The dissected male and female genitalia are preserved in glycerin in small plastic tubes pinned together with the specimens. The distribution map was generated with ARCGIS 10.7.

The type specimens examined are deposited in the Institute of Entomology, Guizhou University, Guiyang, Guizhou Province, China (**IEGU**).

## ﻿Results

### ﻿Taxonomy

**Class Insecta Linnaeus, 1758**

**Order Hemiptera Linnaeus, 1758**

**Infraorder Fulgoromorpha Evans, 1946**

**Family Cixiidae Spinola, 1839**

**Subfamily Cixiinae Spinola, 1839**

**Tribe Pentastirini Emeljanov, 1971**

#### ﻿ Oecleopsis

Taxon classificationAnimaliaHemipteraCixiidae

Emeljanov, 1971

F0788C6F-D53B-5943-9C2D-7B5CFF867ABC

Oecleopsis Emeljanov, 1971: 621; Anufriev and Emeljanov 1988: 460; Van Stalle 1991: 20; Guo et al. 2009: 46; Zhi et al. 2018: 3.

##### Type species.

*Oliarusartemisiae* Matsumura, 1914, original designation.

##### Diagnosis.

For the diagnosis of *Oecleopsis* see Van Stalle (1991: 20) and Guo et al. (2009: 46).

##### Distribution.

**Figure 1.**
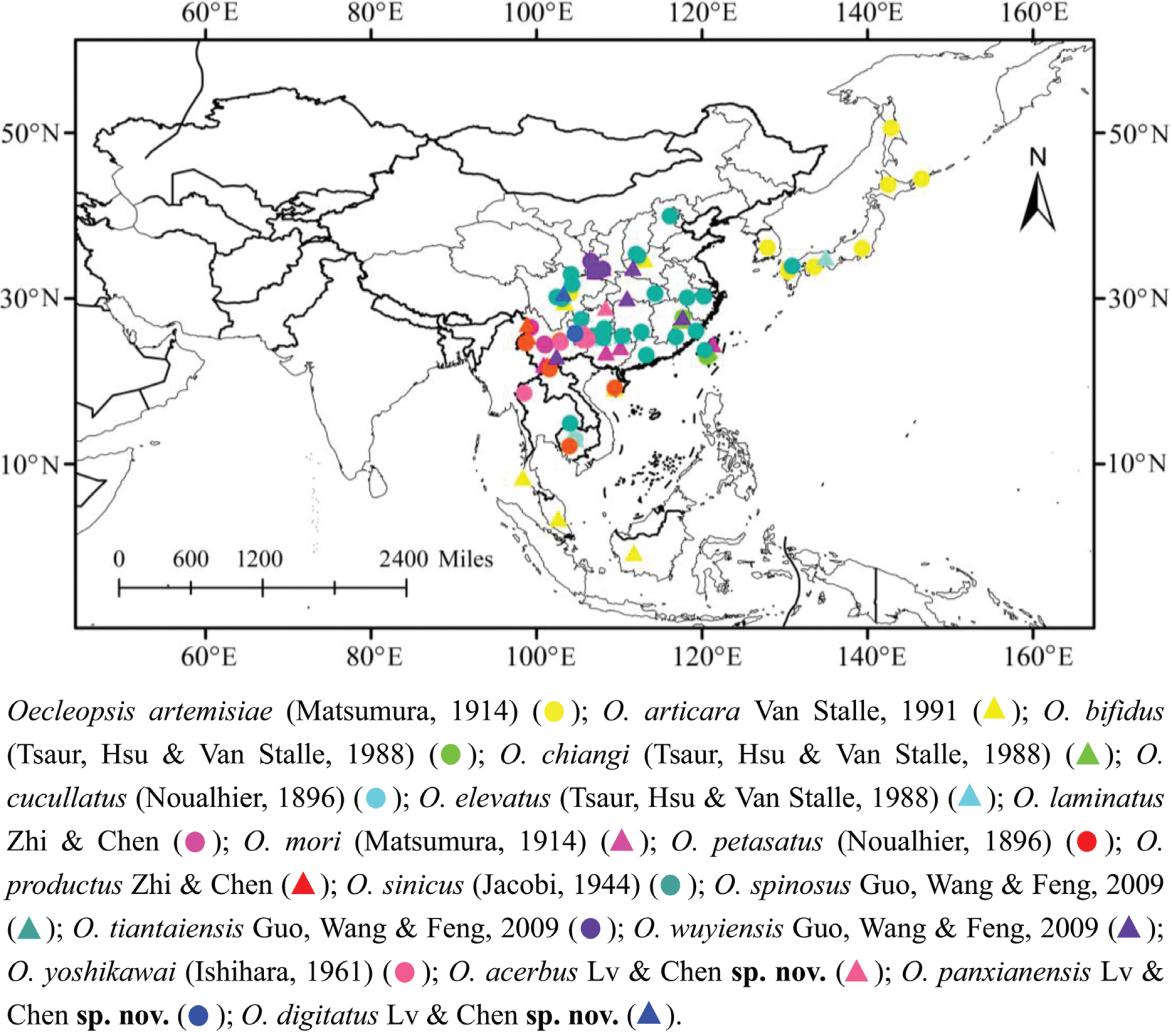
Geographic distributions of *Oecleopsis* species.

China, Japan, Korea, Russia, Thailand, Malaysia, Indonesia and Cambodia (Fig. 1).

### ﻿Checklist and distributions of species of *Oecleopsis* Emeljanov, 1971

*O.artemisiae* (Matsumura, 1914); China (Sichuan Province), Japan (Chishima, Hokkaido, Honshu, Kyushu, Shikoku, Tsushima Islands), Korea, Russia (Kunashir Island).

*O.articara* Van Stalle, 1991; China (Hainan, Henan, Sichuan, Guizhou Provinces), Indonesia (Borneo State), Malaysia (Borneo, Pahang States).

*O.bifidus* (Tsaur, Hsu & Van Stalle, 1988); China (Fujian, Taiwan Provinces).

*O.chiangi* (Tsaur, Hsu & Van Stalle, 1988); China (Fujian, Taiwan Provinces).

*O.cucullatus* (Noualhier, 1896); China (Guangdong, Hubei Provinces), Cambodia.

*O.elevatus* (Tsaur, Hsu & Van Stalle, 1988); China (Guizhou, Taiwan Provinces, Guangxi Zhuang Autonomous Region), Japan (Honshu Island).

*O.laminatus* Zhi & Chen, 2018; China (Yunnan Province).

*O.mori* (Matsumura, 1914); China (Guangxi Zhuang Autonomous Region, Yunnan, Taiwan Provinces).

*O.petasatus* (Noualhier, 1896); China (Hainan, Sichuan, Yunnan Provinces), Cambodia.

*O.productus* Zhi & Chen, 2018; China (Yunnan Province).

*O.sinicus* (Jacobi, 1944); China (Anhui, Beijing, Fujian, Guangdong, Guizhou, Hubei, Henan, Hunan, Shanxi, Sichuan, Zhejiang, Taiwan Provinces, Guangxi Zhuang Autonomous Region), Cambodia, Japan (Kyushu Island).

*O.spinosus* Guo, Wang & Feng, 2009; China (Shaanxi Province).

*O.tiantaiensis* Guo, Wang & Feng, 2009; China (Gansu, Shaanxi Provinces).

*O.wuyiensis* Guo, Wang & Feng, 2009; China (Fujian, Henan, Hunan, Shaanxi, Yunnan Provinces).

*O.yoshikawai* (Ishihara, 1961); China (Guizhou, Yunnan Provinces), Thailand (Doi Inthanon National Park).

*O.acerbus* Lv & Chen sp. nov.; China (Guizhou Province).

*O.panxianensis* Lv & Chen sp. nov.; China (Guizhou Province).

*O.digitatus* Lv & Chen sp. nov.; China (Sichuan Province).

### ﻿Key to species of *Oecleopsis* Emeljanov, 1971

**Table:** 

1	Vertex at least three times as long as broad	**2**
–	Vertex less than three times as long as broad	**6**
2	Apex of endosoma circular	**3**
–	Apex of endosoma not circular	**4**
3	Left side near apex of periandrium with a short spinose process; dorsal margin of endosoma with a long spinose process and left side with a short process (Zhi et al. 2018: figs 31–34)	***O.productus***
–	Left side of periandrium without spinose process; dorsal margin of endosoma without process and left side with a long process (Van Stalle 1991: fig. 79)	***O.articara***
4	Aedeagus with four processes in total (Van Stalle 1991: fig. 72)	***O.petasatus***
–	Aedeagus with three processes in total	**5**
5	Apical process of endosoma bifurcated at basal part, rami long (Fig. 3K–N)	***O.panxianensis* sp. nov.**
–	Apical process of endosoma bifurcated at apical part, rami short relatively (Zhi et al. 2018: figs 17–20)	***O.laminatus***
6	Apical process of endosoma not bifurcated	**7**
–	Apical process of endosoma bifurcated	**10**
7	Apex of endosoma with four processes (Tsaur et al. 1988: fig. 10F, G)	***O.elevatus***
–	Apex of endosoma without four processes	**8**
8	Left side of periandrium at apex of aedeagus with a short spine (Zhi et al. 2018: fig. 46)	***O.yoshikawai***
–	Left side of periandrium at apex of aedeagus without a spine	**9**
9	Periandrium with a moderately long spine, situated on right-dorsal margin, directed dorsocephalad (Tsaur et al. 1988: fig. 8C, D)	***O.chiangi***
–	Periandrium with a short spine, situated on right side of periandrium at apex of aedeagus, directed ventrad (Guo et al. 2009: figs 10, 11)	***O.spinosus***
10	Rami of bifurcation symmetrical, almost equal in length	**11**
–	Rami of bifurcation asymmetrical, unequal in length	**13**
11	Endosoma with one subapical process (Tsaur et al. 1988: fig. 9C, D)	***O.bifidus***
–	Endosoma with two subapical processes	**12**
12	Spine on right side of periandrium at apex of aedeagus very long and slender (Anufriev and Emeljanov 1988: fig. 358; Van Stalle 1991: fig. 101)	***O.artemisiae***
–	Spine on right side of periandrium at apex of aedeagus very short, basal part wide (Van Stalle 1991: fig. 92)	***O.sinicus***
13	Left ramus of bifurcation rudimentary, only a small protuberance (Guo et al. 2009: figs 20, 21)	***O.tiantaiensis***
–	Left ramus of bifurcation well developed	**14**
14	Length of right ramus of bifurcation about three times as long as that of left ramus (Van Stalle 1991: fig. 85)	***O.mori***
–	Length of right ramus of bifurcation less than three times as long as that of left ramus	**15**
15	Ventral margin near base of periandrium with a spinose process (Guo et al. 2009: figs 31, 32)	***O.wuyiensis***
–	Ventral margin near base of periandrium without a spinose process	**16**
16	Dorsal process of endosoma suddenly narrowed at middle part, long, needle-shaped (Fennah 1956: fig. 3 G)	***O.cucullatus***
–	Dorsal process of endosoma not suddenly narrowed at middle part, not long and needle-shaped	**17**
17	Dorsal process of endosoma directed ventrocephalad, lateral margins straight; ventral process constricted in the middle, curved ventrocephalad on left side (Fig. 2K–N)	***O.acerbus* sp. nov.**
–	Dorsal process of endosoma directed dorsocephalad, lateral margins slightly curved; ventral process smoothly tapering at end, curved ventrad on left side (Fig. 4K–N)	***O.digitatus* sp. nov.**

#### ﻿ Oecleopsis acerbus

Taxon classificationAnimaliaHemipteraCixiidae

Lv & Chen sp. nov.

CAE58918-A699-564B-AB2B-6F78D8AC9029

https://zoobank.org/754684CF-A1BB-4257-8655-A714390309A9

Figs 2A–N , 5A–C

##### Type materials.

***Holotype***: China • ♂; Guizhou Province, Yanhe County, Xinjing Town; 28°53'N, 108°17'E; sweeping, 7 June 2007; Pei Zhang leg.; IEGU. ***Paratypes***: 7♂♂, 3♀♀; China • Guizhou Province, Yanhe County, Xinjing Town; 28°53'N, 108°17'E; sweeping, 7 June 2007; Zheng-Guang Zhang & Pei Zhang leg.; IEGU.

##### Diagnosis.

**Figure 2.**
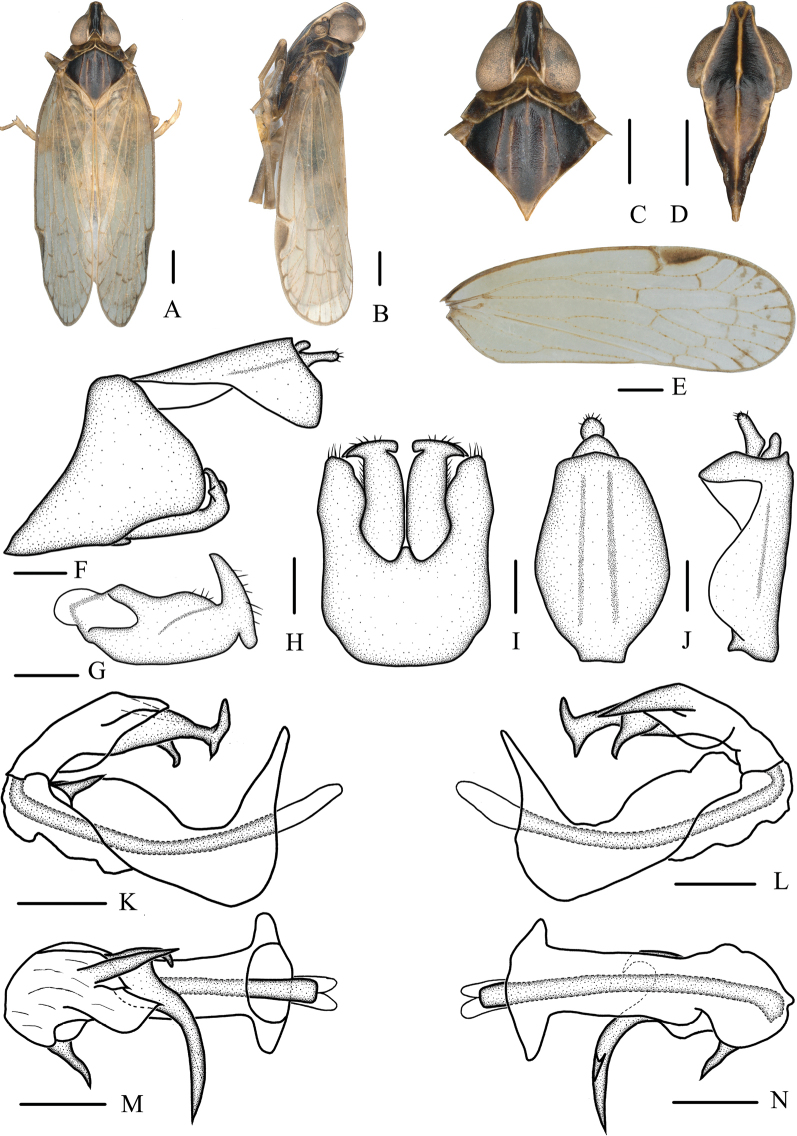
**A–N***Oecleopsisacerbus* sp. nov., male **A** habitus, dorsal view **B** habitus, lateral view **C** head and thorax, dorsal view **D** frons, ventral view **E** forewing **F** male terminalia, lateral view **G** gonostyli, inner lateral view **H** pygofer and gonostyli, ventral view **I** anal segment, dorsal view **J** anal segment, right lateral view **K** aedeagus, right side **L** aedeagus, left side **M** aedeagus, dorsal view **N** aedeagus, ventral view. Scale bars: 0.2 mm.

The salient features of the new species include: vertex (Fig. 2A, C) less than three times as long as broad; spinose process near apex of periandrium on right side (Fig. 2K) short, nib-like; left side (Fig. 2L) apical process of endosoma bifurcated, rami of bifurcation asymmetrical; two subapical spines, dorsal process long and tapering to apex, lateral margins straight, ventral process constricted in the middle.

##### Measurements.

Total length: male 7.20–7.76 mm (*n* = 8), female 7.61–8.05 mm (*n* = 3).

##### Description.

***Coloration*.** General color grayish brown (Fig. 2A, B). Vertex blackish brown. Eyes yellowish brown, ocelli yellowish. Frons yellowish to blackish brown, carinae lighter; rostrum blackish brown. Pronotum blackish brown with carinae yellowish or light brown. Mesonotum blackish brown, carinae brown. Forewings semitranslucent, light grayish brown, distal part with several small patches, stigma yellowish brown. Hindwings semitransparent. Abdomen yellowish brown.

***Head and thorax*.** Vertex (Fig. 2A, C) narrow, 2.36 times longer than wide. Frons (Fig. 1D) with distinct median carina, longer in middle line than wide at widest portion (about 1.33:1). Clypeus (Fig. 2D) with distinct median and lateral carinae. Rostrum elongate, surpassing hind-coxae. Pronotum (Fig. 2C) wider than maximum width of head (including eyes) (1.30:1), lateral and median carinae present. Mesonotum (Fig. 2C) about 6.41 times longer than pronotum in midline, with 5 carinae, distal part of median carina blurry. Forewings (Fig. 2E) slender, longer than maximal width (2.85:1), with 12 apical and 6 subapical cells; fork ScP+R distal to fork CuA_1_+CuA_2_; RP 3 branches, MP_1+2_ 3 branches, and MP_3+4_ 2 branches.

***Male terminalia*.** Pygofer (Fig. 2F, H) symmetrical, in lateral view, lateral lobes triangularly extended caudally; in ventral view, dorsal margin concave and U-shaped. Medioventral process triangular in ventral view. Anal segment (Fig. 2F, I, J) tubular, asymmetrical, widened towards apex in left side view; in right side view, left ventral margin convex and right ventral margin excavated near apex; 1.55 times longer than wide in dorsal view; anal style finger-like, beyond anal tube. Aedeagus (Fig. 2K–N) in total with 4 processes; spinose process near apex of periandrium on right side short relatively, nib-like, directed right-dorsocephalad, only a quarter length of periandrium; left side apical process of endosoma bifurcated, curved outward, rami of bifurcation asymmetrical, dorsal ramus longer and thicker, ventral ramus slender and shorter; 2 subapical spines, dorsal process long and tapering to apex, lateral margins straight, directed ventrocephalad; ventral process constricted in the middle, curved ventrocephalad. Gonostyli (Fig. 2F–H) slender, curved apically, tapering into a process, T-shaped in ventral view.

***Female terminalia*.** Terminalia as shown in Fig. 5A ventrally. Anal segment (Fig. 5C) 1.79 times longer than wide in dorsal view. Posterior vagina (Fig. 5B) elongate, with 4 sclerites in total, dorsal sclerite tapering at the end, median and ventral sclerites long, oval and trapezoidal, respectively, left side of terminal sclerite twist into angular process with straight lateral margins.

##### Distribution.

China (Guizhou) (Fig. 1).

##### Etymology.

The species name is derived from the Latin adjective “*acerbus*”, referring to dorsal process of the endosoma which is pointed on the left side.

##### Remarks.

This species is similar to *Oecleopsiswuyiensis* Guo, Wang & Feng, 2009, but differs from the latter in: (1) frons yellowish to blackish brown (frons black in *O.wuyiensis*); (2) ventral margin near base of periandrium without a spinose process (ventral margin near base of periandrium with a spinose process in *O.wuyiensis*); (3) dorsal process of endosoma directed ventrocephalad (dorsal process of endosoma directed dorsocephalad in *O.wuyiensis*); (4) ventral process of endosoma curved and rounded at apex (ventral process of endosoma straight and pointed in *O.wuyiensis*).

#### ﻿ Oecleopsis panxianensis

Taxon classificationAnimaliaHemipteraCixiidae

Lv & Chen sp. nov.

B939D1BA-4DBB-5034-80F4-56BD243971AD

https://zoobank.org/4A4CF424-4975-44BC-8605-DBA344D63753

Fig. 3A–N

##### Type materials.

***Holotype***: China • ♂; Guizhou Province, Panxian County, Banqiao Town; 25°44'N, 104°39'E; sweeping, 2 July 2011; Zhi-Hua Fan leg.; IEGU. ***Paratype***: 1♂, same collection data as for holotype; IEGU.

##### Diagnosis.

**Figure 3.**
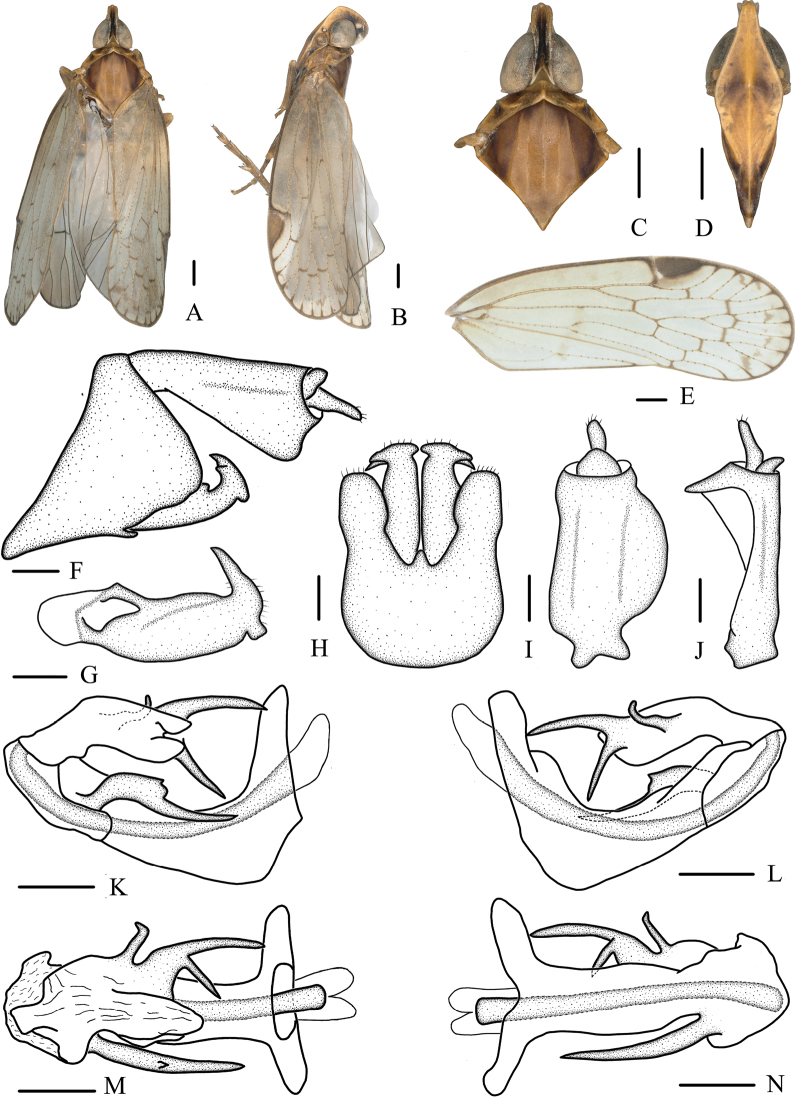
**A–N***Oecleopsispanxianensis* sp. nov., male **A** habitus, dorsal view **B** habitus, lateral view **C** head and thorax, dorsal view **D** frons, ventral view **E** forewing **F** male terminalia, lateral view **G** gonostyli, inner lateral view **H** pygofer and gonostyli, ventral view **I** anal segment, dorsal view **J** anal segment, right lateral view **K** aedeagus, right side **L** aedeagus, left side **M** aedeagus, dorsal view **N** aedeagus, ventral view. Scale bars: 0.2 mm.

The salient features of the new species include: vertex (Fig. 3A, C) at least three times as long as broad; frons (Fig. 3D) yellowish brown, with brown spots; aedeagus (Fig. 3K–N) in total with 3 processes; spinose process near apex of periandrium on right side (Fig. 3K) long, half wide at base, half thinner at end; left side (Fig. 3L) apical process of endosoma bifurcated, rami long, approximately equal length; a subapical spine, lateral margins curved.

##### Measurements.

Total length: male 7.23–7.65 mm (*n* = 2).

##### Description.

***Coloration*.** General color dark brown (Fig. 3A, B). Vertex blackish brown. Eyes brown, ocelli yellowish. Frons yellowish brown, with brown spots, carinae lighter; rostrum blackish brown. Pronotum grayish to blackish brown with carinae yellowish or light brown. Mesonotum dark brown, carinae brown. Forewings semitranslucent, light brown, with several brown markings, stigma yellowish brown. Hindwings semitransparent. Abdomen dark brown.

***Head and thorax*.** Vertex (Fig. 3A, C) narrow, 3.23 times longer than wide. Frons (Fig. 3D) with distinct median carina, longer in middle line than wide at widest portion (about 1.67:1). Clypeus (Fig. 3D) with distinct median and lateral carinae. Rostrum elongate, surpassing hind-coxae. Pronotum (Fig. 3C) wider than maximum width of head (including eyes) (1.60:1), lateral and median carinae present. Mesonotum (Fig. 3C) about 6.92 times longer than pronotum in midline, with 5 carinae, distal part of median carina blurry. Forewings (Fig. 3E) slender, longer than maximal width (3.01:1), with 12 apical and 6 subapical cells; fork ScP+R distal to fork CuA_1_+CuA_2_; RP 3 branches, MP_1+2_ 3 branches, and MP_3+4_ 2 branches.

***Male terminalia*.** Pygofer (Fig. 3F, H) symmetrical, in lateral view, lateral lobes triangularly extended caudally; in ventral view, dorsal margin concave and U-shaped, middle part narrow. Medioventral process triangular in ventral view. Anal segment (Fig. 3F, I, J) tubular, asymmetrical, widened towards apex in left side view; in right side view, left ventral margin convex and right ventral margin excavated near apex; 1.68 times longer than wide in dorsal view; anal style finger-like, beyond anal tube. Aedeagus (Fig. 3K–N) in total with 3 processes; spinose process near apex of periandrium on right side long, half wide at base, half thinner at end, directed cephalically, more than 1/2 length of periandrium; left side apical process of endosoma bifurcated, rami long, approximately equal length, dorsal ramus directed cephalad, ventral ramus directed ventrocephalad; a subapical spine, lateral margins curved, curved dorsad, apical margin rounded. Gonostyli (Fig. 3F–H) slender, curved apically, tapering into a process, constricted for about ¾ its length, T-shaped in ventral view.

##### Distribution.

China (Guizhou) (Fig. 1).

##### Etymology.

The new species is named after its the county in which it was collected.

##### Remarks.

This species is similar to *Oecleopsislaminatus* Zhi & Chen, 2018, but differs from the latter in: (1) spinose process near apex of periandrium long, half wide at the base, ½ thinner at end (spinose process near apex of periandrium long, smoothly tapering at the end in *O.laminatus*); (2) apical process of endosoma bifurcated at base (apical process of endosoma bifurcated at apex in *O.laminatus*); (3) left side near apex of endosoma with a spiniform process, curved dorsad (left side near apex of endosoma with a large laminal process, directed cephalad in *O.laminatus*).

#### ﻿ Oecleopsis digitatus

Taxon classificationAnimaliaHemipteraCixiidae

Lv & Chen sp. nov.

9DC142F6-D85E-5717-85FB-990C01B306D7

https://zoobank.org/49B9BA46-A908-44F6-8CD8-92A1373CF46C

Figs 4A–N , 5D–F

##### Type materials.

***Holotype***: China • ♂; Sichuan Province, Dayi County, Xiling Town; 30°38'N, 103°14'E; sweeping, 20 July 2022; Sha-Sha Lv leg.; IEGU. ***Paratypes***: China • 6♂♂4♀♀; Sichuan Province, Dayi County, Xiling Town; 30°38'N, 103°14'E; sweeping, 20 July 2022; Sha-Sha Lv, Lan Zhang, Yong-Jin Sui & Feng-E Li leg.; IEGU.

##### Diagnosis.

**Figure 4.**
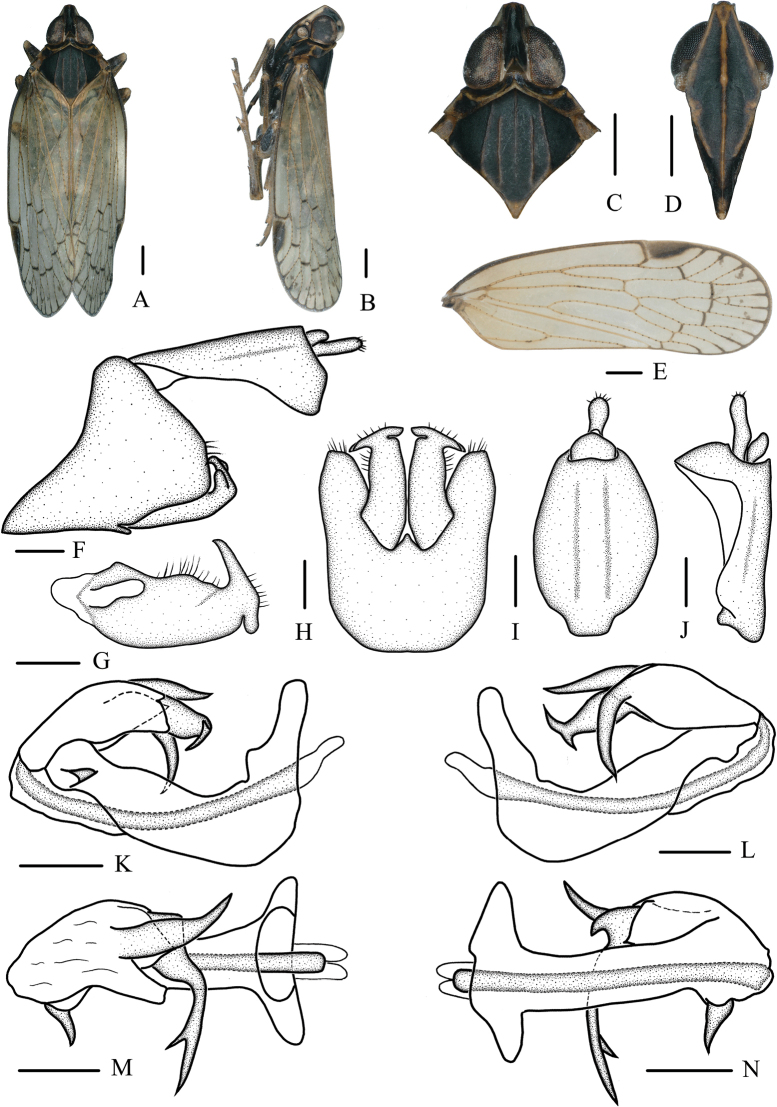
**A–N***Oecleopsisdigitatus* sp. nov., male **A** habitus, dorsal view **B** habitus, lateral view **C** head and thorax, dorsal view **D** frons, ventral view **E** forewing **F** male terminalia, lateral view **G** gonostyli, inner lateral view **H** pygofer and gonostyli, ventral view **I** anal segment, dorsal view **J** anal segment, right lateral view **K** aedeagus, right side **L** aedeagus, left side **M** aedeagus, dorsal view **N** aedeagus, ventral view. Scale bars: 0.2 mm.

The salient features of the new species include: vertex (Fig. 4A, C) less than three times as long as broad; spinose process near apex of periandrium on right side (Fig. 4K) short and thick, finger-like; left side (Fig. 4L) apical process of endosoma bifurcated, rami short; two subapical spines long, dorsal process tapering toward the end, ventral process curved.

##### Measurements.

Total length: male 5.8–6.4 mm (*n* = 7), female 6.8–7.5 mm (*n* = 5).

##### Description.

***Coloration*.** General color grayish black (Fig. 4A, B). Vertex brown-black. Eyes dark brown, ocelli yellowish. Frons black, carinae yellowish brown; rostrum black. Pronotum brown-black with carinae yellowish or light brown. Mesonotum black, carinae brown. Forewings semitranslucent, yellowish brown, with several small brown markings, stigma brown. Hindwings semitransparent. Abdomen dark brown.

***Head and thorax*.** Vertex (Fig. 4A, C) narrow, 2.41 times longer than wide. Frons (Fig. 4D) with distinct median carina, longer in middle line than wide at widest portion (about 1.45:1). Clypeus (Fig. 4D) with distinct median and lateral carinae. Rostrum elongate, surpassing hind-coxae. Pronotum (Fig. 4C) wider than maximum width of head (including eyes) (1.39:1), lateral and median carinae present. Mesonotum (Fig. 4C) about 7.17 times longer than pronotum in midline, with 5 carinae, distal part of median carina blurry. Forewings (Fig. 4E) slender, longer than maximal width (2.94:1), with 12 apical and 6 subapical cells; fork ScP+R distal to fork CuA_1_+CuA_2_; RP 3 branches, MP_1+2_ 3 branches, and MP_3+4_ 2 branches.

***Male terminalia*.** Pygofer (Fig. 4F, H) symmetrical, in lateral view, lateral lobes triangularly extended caudally; in ventral view, dorsal margin concave and U-shaped, widened towards apex. Medioventral process triangular in ventral view. Anal segment (Fig. 4F, I, J) tubular, asymmetrical, widened towards apex in left side view; in right side view, left ventral margin convex and right ventral margin excavated near apex; 1.64 times longer than wide in dorsal view; anal style finger-like, beyond anal tube. Aedeagus (Fig. 4K–N) with 4 processes in total; spinose process near apex of periandrium on right side short and thick, directed dorsocephalad, finger-like, only a 1/4 length of periandrium; left side apical process of endosoma bifurcated, curved outward, rami short, dorsal ramus directed dorsad, ventral ramus directed ventrad; 2 subapical spines long, dorsal process tapering toward end, directed cephalad; ventral process curved, curved ventrad. Gonostyli (Fig. 4F–H) slender, curved apically, tapering into a process, curved portion near right angle, T-shaped in ventral view.

**Figure 5.**
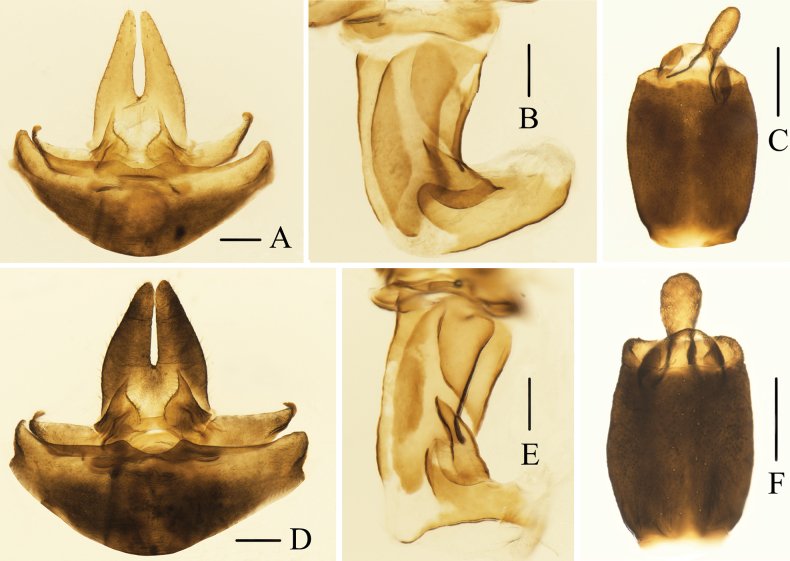
**A–F** female terminalia of *Oecleopsis* species **A–C***Oecleopsisacerbus* sp. nov. **D–F***Oecleopsisdigitatus* sp. nov. **A, D** female terminalia, ventral view **B, E** posterior vagina, ventral view **C, F** anal segment, dorsal view. Scale bars: 0.2 mm.

***Female terminalia*.** Terminalia, viewed ventrally, are shown in Fig. 5D. Anal segment (Fig. 5F) 1.84 times longer than wide in dorsal view. Posterior vagina (Fig. 5E) elongate, with 4 sclerites in total, dorsal sclerite tapering at the end, median and ventral sclerites long, suboblong and slender, respectively, left side of terminal sclerite twist into thick finger-like process.

##### Distribution.

China (Sichuan) (Fig. 1).

##### Etymology.

The species name is derived from the Latin adjective “*digitatus*”, referring to the finger-like spinose process near apex of periandrium on the right side.

##### Remarks.

This species is similar to *Oecleopsissinicus* (Jacobi, 1944), but differs from the latter in: (1) MP_1+2_ of forewing 3 branches (MP_1+2_ of forewing 2 branches in *O.sinicus*); (2) rami of bifurcation asymmetrical, unequal in length (rami of bifurcation symmetrical, equal in length in *O.sinicus*); (3) spinose process near apex of periandrium finger-like on right side, directed dorsocephalad (spinose process near apex of periandrium awl-shaped on right side, directed ventrocephalad in *O.sinicus*).

## ﻿Discussion

Host plant information is less well documented in cixiids, especially in the genus *Oecleopsis*, where the host plants of only two species have been recorded so far. *Oecleopsissinicus* (Jacobi, 1944) was collected on *Artemisia* L. sp. (Asteraceae) and *Zeamays* L. (Panicoideae), and *O.yoshikawai* (Ishihara, 1961) was collected on bamboo (Bambusoideae) (Zhi et al. 2018). Since these two discoveries, no additional information has been found on the ecology or behavior of any other species of *Oecleopsis*.

Based on data from published information and our field surveys, the distribution of *Oecleopsis* is restricted to the Palaearctic and Oriental regions (Fig. 1) (Holt et al. 2013), and the genus especially speciose in China where all species have been recorded to date. However, at present, it is mainly known to occur in Central, East, South, and Southwest China, but collection and survey data are still not comprehensive enough. Therefore, we believe that additional wide-ranging field surveys will find that the diversity of *Oecleopsis* in China is doubtlessly richer.

## Supplementary Material

XML Treatment for Oecleopsis

XML Treatment for Oecleopsis acerbus

XML Treatment for Oecleopsis panxianensis

XML Treatment for Oecleopsis digitatus

## ﻿Additional information

### Author ORCIDs

Sha-Sha Lv https://orcid.org/0000-0001-5353-5082

Lin Yang https://orcid.org/0000-0002-7841-5156

Yu-Bo Zhang https://orcid.org/0000-0002-6118-6190

Yan Zhi https://orcid.org/0000-0003-1826-8139

Pei Zhang https://orcid.org/0009-0009-0251-0980

Xiang-Sheng Chen https://orcid.org/0000-0001-9801-0343

### Data availability

All of the data that support the findings of this study are available in the main text.
